# A randomised controlled trial of sensory awareness training and additional motor practice for learning scalpel skills in podiatry students

**DOI:** 10.1186/s12909-016-0817-8

**Published:** 2016-12-05

**Authors:** Ryan S Causby, Michelle N McDonnell, Lloyd Reed, Susan L Hillier

**Affiliations:** 1 Sansom Institute for Health Research, University of South Australia, GPO Box 2471, Adelaide, SA 5001 Australia; 2 Institute for Health and Biomedical Innovation, Queensland University of Technology, Brisbane, Australia

**Keywords:** Dexterity, Scalpel skills, Clinical Skills, Motor learning, Psychomotor testing, Purdue Pegboard, Grooved Pegoard Test, Grip-lift test, Intrinsic Motivation Inventory

## Abstract

**Background:**

The process of using a scalpel, like all other motor activities, is dependent upon the successful integration of afferent (sensory), cognitive and efferent (motor) processes. During learning of these skills, even if motor practice is carefully monitored there is still an inherent risk involved. It is also possible that this strategy could reinforce high levels of anxiety experienced by the student and affect student self-efficacy, causing detrimental effects on motor learning. An alternative training strategy could be through targeting sensory rather than motor processes.

**Methods:**

Second year podiatry students who were about to commence learning scalpel skills were recruited. Participants were randomly allocated into sensory awareness training (Sensory), additional motor practice (Motor) or usual teaching only (Control) groups. Participants were then evaluated on psychological measures (Intrinsic Motivation Inventory) and dexterity measures (Purdue Pegboard, Grooved Pegboard Test and a grip-lift task).

**Results:**

A total of 44 participants were included in the study. There were no baseline differences or significant differences between the three groups over time on the Perceived Competence, Effort/ Importance or Pressure/ Tension, psychological measures. All groups showed a significant increase in Perceived Competence over time (F_1,41_ = 13.796, *p* = 0.001). Only one variable for the grip-lift task (Preload Duration for the non-dominant hand) showed a significant difference over time between the groups (F_2,41_ = 3.280, *p* = 0.038), specifically, Motor and Control groups.

**Conclusions:**

The use of sensory awareness training, or additional motor practice did not provide a more effective alternative compared with usual teaching. Further research may be warranted using more engaged training, provision of supervision and greater participant numbers.

**Trial registration:**

Australian New Zealand Clinical Trials Registry (ANZCTR): ACTRN12616001428459. Registered 13^th^ October 2016. Registered Retrospectively.

**Electronic supplementary material:**

The online version of this article (doi:10.1186/s12909-016-0817-8) contains supplementary material, which is available to authorized users.

## Background

Many professions and trades require skilled performance of gross and fine motor skills. The health professions are no exception with many, including podiatry, relying on dextrous use of small tools such as scalpels. Dexterity can be defined as:

*“manual ability that requires rapid coordination of gross or fine voluntary movements, based on a certain number of capacities, which are developed through learning, training and experience”* [1].

The process of using a scalpel, like all other motor activities, is dependent upon the successful integration of a number of systems. Based on the dynamical systems theory (DST), our current belief is that voluntary movement involves the interaction of three systems: afferent (sensory), cognitive and efferent (motor). Integration of these systems is complex and not simply sequential. Using predictive processes, based on previous experiences, a motor plan may be devised and implemented. Whilst the plan is being executed, sensory feedback loops back into the system, enabling a comparison of the resultant and intended plan and subsequent modification as necessary. A practical example of this overall process is when someone intends to lift a milk carton which they may be expecting to be full, but instead is empty. The devised motor plan results in greater force being applied than required, with a subsequent overshooting of the target requirement. At this point visual, tactile and kinaesthetic feedback intervenes to enable motor adjustment and avoid the carton being thrown and milk spilt.

From a series of interviews in Australia and New Zealand with university staff involved in clinical podiatric teaching, there were some common student issues identified in the teaching of psychomotor skills, specifically scalpel skills. This included the high level of anxiety often experienced by students, low levels of self-efficacy and issues with innate dexterity [unpublished observations]. Furthermore, a number of strategies to manage poor-performing students were identified, many of which focus on the motor aspects of the sensorimotor system, with little consideration of afferent and cognitive elements. For example, one of the methods employed to increase the exposure of students to practical scalpel use is by implementing additional practice on colleagues and patients within the clinic setting, or to undertake further scalpel tasks on inanimate objects. Even if practice on live participants is carefully monitored there is still an inherent risk involved. It is also possible that this strategy could reinforce high levels of anxiety experienced by the student and affect student self-efficacy, causing detrimental effects on psychomotor learning. An alternative training strategy could be through targeting sensory rather than motor processes.

One way this may be achieved is through active sensory training. This involves an active engagement of the learner via education, proprioceptive training, the localisation and discrimination of sensation and other sensory practice techniques. Active sensory training, if effective, would have greater feasibility and utility in the scalpel learning context. However, there is little evidence relating to improving hand function or dexterity via sensory training. Two systematic reviews included studies related to sensory training for rehabilitation following stroke, most of which did not evaluate motor learning or dextrous hand function [2, 3].

Only one study evaluated the effects of sensory awareness training compared to sham relaxation sessions on university students using two objective and one self-reported measure of dexterity [4]. Sensory awareness training was implemented via the Feldenkrais Awareness Through Movement (ATM) method. Feldenkrais is “an educational system that develops a functional awareness of the self in the environment” [5] and is linked to DST which has an underlying principle that movement requires the interaction of sensory (perceptual), cognitive and motor systems [6]. This study involved the use of a single group-style intervention focussing on the dominant hand or non-dominant hand, or a sham relaxation session, for 40 min duration with dexterity assessment immediately before and after the lesson. The selected objective tests included the Purdue Pegboard (PP) [7] and the Grip-lift task [8] discussed in detail later. A comparison was made of dexterity of the dominant and non-dominant hands using the PP, Grip-lift task and self-perceived changes. A significant difference was found in favour of sensory awareness training on performance for the PP, and a desirable decrease in maximum grip force on the Grip-lift task, indicating an improvement in dexterity. Self-reported perceived changes found that 100% of the intervention group perceived changes after sensory awareness training, changes between their hands and a decrease in difficulty when performing handwriting, compared with only 50–60% of the control group perceiving differences and no perceived change in hand-writing difficulty. Thus, we considered the possibility that a similar intervention may be successful in promoting dextrous motor learning such as with scalpel skills, including an influence on self-efficacy and anxiety levels.

The aim of this study was to determine via a repeated measures randomised controlled trial whether sensory training or additional motor training would produce dexterous performance superior to a no-intervention control group. We hypothesised that sensory training would be equivalent or better than motor training. Sensory training could then provide a safer alternative to standard teaching or additional motor practice thereby decreasing anxiety, increasing self-efficacy and improving scalpel skill acquisition.

## Methods

Participants were included if they were second year podiatry students from the University of South Australia (UniSA) and the Queensland University of Technology (QUT), about to commence learning scalpel and other related manual skills in February and March 2013. Participants were excluded if they had previously used a scalpel, had an occupation or a hobby which required high levels of dexterity, were on medication or had a condition which could affect hand function. On the day of testing, participants were requested not to undertake vigorous exercise or drink caffeine prior to testing. At the initial appointment demographic, medical, work and hobby information was obtained before participants completed a modified Intrinsic Motivation Inventory (IMI) [9]. Following this, testing was undertaken with the following tests in order: PP, Grooved Pegboard Test (GPT) [10] and the Grip-Lift task. There is a lack of precedent from which to accurately calculate a sample size, however a sample size for 80% power and based on an effect size of 0.75 for the PP in Bitter et al. [4] suggests 29 participants per group. All participants provided written, informed consent, in accordance with the Declaration of Helsinki. Ethics approval was obtained from the University of South Australia and Queensland University of Technology ethics committees (protocol number 0000027836).

### Measures

**Fig. 1:**
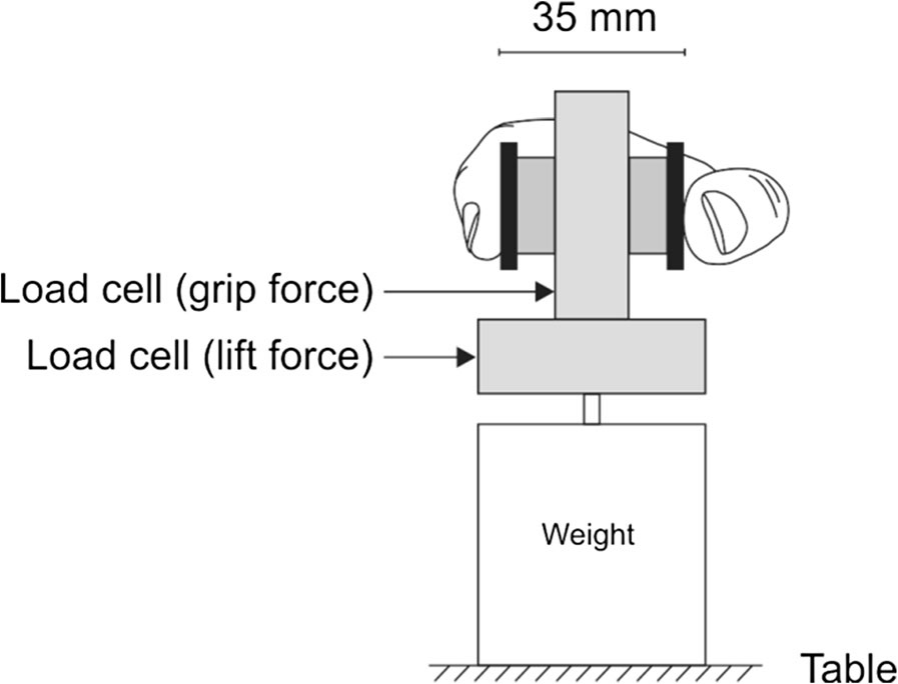
Grip Lift Manipulandum

The IMI contains six validated sub-scales (‘Interest/ Enjoyment’, ‘Perceived Competence’, ‘Effort/ Importance’, ‘Pressure/ Tension’, ‘Value/ Usefulness’ and ‘Perceived Choice’) which can be used individually [11]. Responses to each of the questions are reported on a Likert-style scale from 1 (not at all true) to 7 (very true). We selected the sub-scales ‘Perceived Competence’, ‘Effort/ Importance’ and ‘Pressure/ Tension’ to represent a student’s self-efficacy, motivation and anxiety and modified the questions to represent scalpel use. The IMI can reportedly be used with the inclusion or exclusion of specific subscales without any impact on the others, and with slight modification to the wording to reflect specific tasks without affecting validity [9]. The PP test requires subjects to insert as many pins as possible in a row of holes in 30 s, using the left hand, or right hand. The GPT (model 32025, Lafayette Instrument, Lafayette, IN, USA) has 25 keyhole style slots of varying orientation throughout and like the PP is purported to test psychomotor speed, fine motor control, and rapid visual-motor coordination [12]. Sensory feedback is required to sense the orientation of the groove on the pin prior to placement, and greater coordination is required than the PP [13]. The fastest time recorded for the participant to place all 25 pegs for each hand was used for data analysis. The Grip-Lift task uses a manipulandum, similar to that used by Westling and Johansson [8], for measuring grip force and lift force whilst a small object is gripped and lifted off the supporting surface. As shown in Fig. 1, two linear strain gauges (model MLP-100; Transducer Techniques, Temecula, CA, USA) detect the grip force (horizontal force) applied to the object whilst simultaneously recording lift forces (vertical) as the device is lifted to a pre-determined height. Two brass pads are positioned at the top of the device and sit approximately 35 mm apart. Below the second force gauge there is a metal strip which provides a ledge upon which a variety of weights may be placed to alter the overall weight of the device. This weight is indeterminable to the participant to prevent visual-based prediction of weight whereby they could establish an anticipatory strategy for lifting the manipulandum. To lift the device (three lifts per hand), participants used a pincer-style grip also known as a precision grip [8, 14, 15], similar to the thumb and forefinger grip commonly used to hold a scalpel handle. Methods employed were similar to those reported by McDonnell et al. [16] and Todd et al. [17]. The output signal was amplified (1000x) using a custom amplifier, and filtered (low-pass 100 Hz) and recorded using CED data acquisition hardware 1401and using Spike2 v.7 software (Cambridge Electronic Design, Cambridge, UK). Seven different outcome measures were considered to evaluate dexterity, as previously described [14, 18]. These outcomes included temporal (preload duration, lift duration), force (maximum grip force, average grip force, hold ratio), and variability characteristics (maximum cross-correlation and the standard deviation of the grip force) of the lift and the hold phase. The outcomes of interest are fully described in Additional file 1.

The average values from the second and third lifts for each hand were calculated and exported for statistical analysis. The first lifts were discarded due to a large number of fumbles by participants.

### Groups and training

After baseline testing, prior to initial scalpel skill teaching, participants were randomly allocated into one of three groups by a person independent to the study using a computer-generated list, stratified across sites. The three groups comprised of the additional sensory awareness training (Sensory), additional motor practice (Motor) or usual teaching only (Control). The importance of research rigour was emphasised and participants were requested not to discuss their activities with other study participants from other groups. The principal researcher and assessor (RC) remained blinded to group allocation.

### Sensory awareness training (Sensory)

Participants randomised to the Sensory group were taken into a separate classroom to receive sensory awareness training in the form of Feldenkrais ATM from an experienced trainer (SLH) for approximately 40 min. This was then supplemented with audio recordings of two sessions, a ‘sitting’ session focusing on relaxation and awareness and another session targeting the dominant hand which were provided via CD and a link for downloading the files over the internet if required for use at home.

### Motor practice (Motor)

The Motor group participants were also taken aside immediately and instructed in methods for motor practice in the form of staged scalpel practice including: holding a pen in the same manner as a scalpel and replicating scalpel movement either in free space or on paper varying the applied pressure; using a scalpel handle without a blade attached; and using a scalpel on inanimate objects such as soap or oranges to replicate normal usage on the foot. The QUT cohort was unable to use blades at home due to local policy and were therefore provided with dedicated times when they could undertake additional motor practice with scalpels at the university. They were also encouraged to practice with the scalpel handle without a blade at home.

Participants were encouraged to undertake the training sessions as regularly as they could, a minimum of three times per week in their own time for 2 weeks. Participants in both intervention groups were provided with a diary to record details of their training sessions including the frequency, duration and additional details if required (e.g., which exercises were completed). During the intervention period all participants were sent short message service (SMS) or text messages periodically via skype to their mobile phones as a reminder to undertake their required training if allocated to a training group. After a minimum intervention period of 2 weeks, participants were invited to book for post-intervention testing, the exact period varied for each participant depending on when the follow-up booking could be made. They completed the IMI and the objective tests used in the original testing battery using the same protocols as previously outlined, facilitated by the same assessor (RC) who reiterated to participants that he was blinded to group allocation.

### Analysis

Continuous and categorical group characteristics were compared statistically using SPSS v.21 (IBM Corporation, Armonk, NY, USA) computer software. Categorical values such as location, sex, handedness, musical instrument history and gaming history were compared using a Chi Square analysis. Continuous variables such as age, height and intervention duration (total time) were compared using a generalised linear model univariate analysis of variance. Data were evaluated for normal distribution and transformed as necessary. IMI sub-categories were compared using repeated measures mixed models analysis with location included as a fixed effect to account for possible variation in standard teaching or intervention implementation between sites. The objective dexterity tests were also compared for between group differences using repeated measures mixed models analysis. Age, sex, location, handedness and other demographic variables were either included or removed as fixed variables to determine the most appropriate model based on Bayesian Information Criterion (BIC) values (the lowest value indicating the best model [19]). Estimated marginal means were reported (rather than raw means) for the model, as these were the means used for comparison and are calculated by taking into account the effect of any other variables used in the model. Significance was set at *p* < 0.05.

## Results

**Fig. 2:**
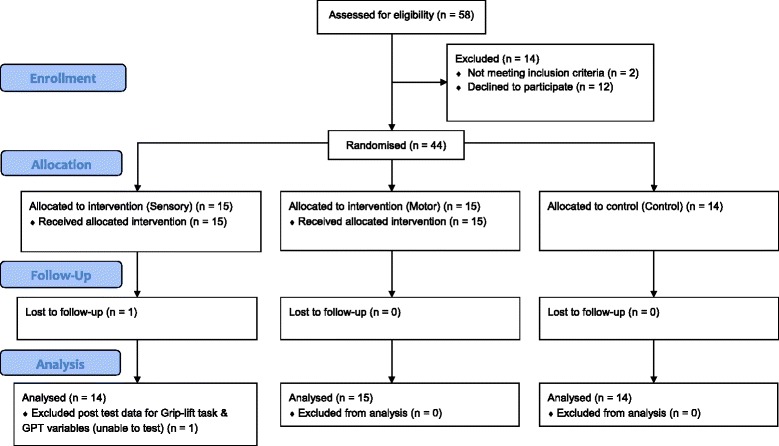
CONSORT Flow diagram

A total of 44 participants were included in the study (Fig. 2); 29 (65.9%) were female and 15 (34.1%) male; 84.1% were right handed. Group characteristics were balanced for all descriptive characteristics except for computer gaming history where the control group had a significantly greater proportion with a gaming history. Details can be found in Table 1 below.

**Table 1:** Group demographic characteristics

	Control (*n* = 14)	Sensory (*n* = 15)	Motor (*n* = 15)	*p*-value
Location, n (%)				
UniSA	7 (50%)	7 (46.7%)	7 (46.7%)	0.94
QUT	7 (50%)	8 (53.3%)	8 (53.3%)	
Sex, n (%)				
Male	7 (50%)	4 (26.7%)	4 (26.7%)	0.32
Female	7 (50%)	11 (73.3%)	11 (73.3%)	
Age (years), Mean (SD)	22.4 (6.5)	23.4 (7.8)	22.9 (6.2)	0.93
Handedness, n (%)				
Right	12 (85.7%)	12 (80.0%)	13 (86.7%)	0.84
Left	1 (7.1%)	2 (13.3%)	2 (13.3%)	
Ambidextrous	1 (7.1%)	1 (6.7%)	0 (0%)	
Height (cm), Mean (SD)	172.1 (12.6)	168.7 (7.1)	167.5 (11.2)	0.49
Musical History, n (%)				
Yes	3 (21.4%)	1 (6.7%)	2 (13.3%)	0.51
No	11 (78.6%)	14 (93.3%)	13 (86.7%)	
Gaming History, n (%)				
Yes	7 (50%)	1 (6.7%)	2 (13.3%)	0.01*
No	7 (50%)	14 (93.3%)	13 (86.7%)	

*p ≤ 0.05 Chi square used to calculate *p*-values for categorical variables and univariate analysis for continuous variables

### Intervention and data testing compliance

One participant (sensory group) was lost to follow-up due to health reasons not related to the study. A second participant (sensory group) did not return their intervention diary recording practice duration and withdrew from the podiatry program shortly after post-intervention testing. The mixed model statistical method allows any data collected on participants lost to follow-up to remain in the analysis and these data were therefore retained.

There was no significant difference between the two intervention groups for intervention duration, mean practice time or number of sessions (see Table 2).

**Table 2:** Mean intervention, practice sessions and practice times, and *p*-values

	Control (*n* = 14)	Sensory (*n* = 15)	Motor (*n* = 15)	*p*-value
Intervention Duration (days)	48.14 (21.25)	49.50 (22.39)	53.36 (22.86)	0.813
Practice sessions (n)	-	6.92 (3.52)	8.80 (3.12)	0.147
Practice time (overall) (min)	-	293.23 (186.00)	221.20 (97.86)	0.195

*p*-values calculated by univariate analysis

There were no significant differences between the three groups over time on Perceived Competence, Effort/Importance or Pressure/Tension, as shown in Table 3. All groups showed a significant increase in Perceived Competence (F_1,41_ = 13.796, *p* = 0.001) during the intervention period. The motor group showed the greatest increase in Perceived Competence but not significantly more than the other groups. There was no significant change in Effort/ Importance (F_1,39_ = 0.006, *p* = 0.939) or Pressure/ Tension (F_1,41_ = 0.447, *p* = 0.507).

**Table 3:** Raw means pre and post intervention and *p*-values for Intrinsic Motivation Inventory (IMI)

Means (SD)	Control		Sensory		Motor		F value	*P*-value (Group x Time)
	(*n* = 14)		(*n* = 15)		(*n* = 15)			
	*Pre*	*Post*	*Pre*	*Post*	*Pre*	*Post*		
Perceived Competence^a^	22.79 (5.63)	25.50 (3.59)	23.20 (7.43)	26.43 (6.78)	25.71 (6.57)	30.07 (5.78)	0.17	0.84
Effort / Importance	30.07 (3.45)	29.00 (4.24)	29.00 (5.21)	29.50 (4.75)	28.86 (4.64)	29.27 (5.26)	0.70	0.50
Pressure / Tension	21.64 (6.80)	22.50 (5.17)	20.07 (5.36)	20.21 (6.95)	20.50 (4.60)	17.67 (5.15)	1.62	0.21

^a^Model includes ‘history of computer gaming’ variable as a random effect

*p*-values calculated using linear mixed model analysis

A linear mixed model analysis of the three dexterity tests showed that only one variable, Preload Duration for the non-dominant hand, showed a significant group difference over time (F_2,41_ = 3.280, *p* = 0.038) (Table 4). This was calculated on Log_10_ transformed data to address the skewed distribution of data. There were no other significant differences between the three groups over time.

**Table 4:** Raw means pre and post intervention values and *p*-values for dexterity measures

Task	Outcomes	Means (SD)						F value	*P*-value (Grp x Time)
		Control (*n* = 14)		Sensory (*n* = 15)		Motor (*n* = 15)			
		*Pre*	*Post*	*Pre*	*Post*	*Pre*	*Post*		
Grip-Lift task	Preload duration (ms)								
	Dominant	176.22 (180.15)	123.37 (42.56)	98.08 (35.21)	98.58 (36.40)	157.66 (228.74)	129.13 (62.24)	0.12	0.89
	Non-dominant	181.21 (137.66)	120.00 (65.84)	72.00 (48.82)	88.44 (40.14)	90.71 (53.36)	131.20 (79.88)	3.53	0.04*
	Maximum grip force (N)								
	Dominant	5.07 (1.99)	4.57 (1.79)	6.52 (2.89)	6.55 (2.81)	7.22 (4.73)	7.13 (3.55)	0.19	0.83
	Non-dominant	6.18 (2.57)	5.34 (2.10)	6.80 (2.67)	7.71 (6.11)	7.67 (4.27)	7.59 (3.13)	0.67	0.52
	Maximum correlation (ρ)								
	Dominant	0.77 (0.11)	0.81 (0.06)	0.77 (0.07)	0.78 (0.06)	0.78 (0.07)	0.75 (0.10)	0.35	0.71
	Non-dominant	0.77 (0.07)	0.78 (0.07)	0.76 (0.07)	0.77 (0.07)	0.78 (0.06)	0.77 (0.07)	0.47	0.63
	Average Grip (N)								
	Dominant	4.48 (1.75)	3.69 (1.27)	5.47 (3.11)	5.16 (3.14)	6.10 (3.81)	5.63 (2.68)	0.40	0.68
	Non-dominant	4.49 (1.92)	3.97 (1.55)	5.23 (2.07)	5.77 (5.54)	5.30 (2.46)	6.00 (2.73)	0.55	0.58
	SD Grip								
	Dominant	0.13 (0.07)	0.10 (0.06)	0.18 (0.13)	0.16 (0.17)	0.17 (0.11)	0.14 (0.12)	0.45	0.64
	Non-dominant	0.16 (0.07)	0.13 (0.09)	0.16 (0.07)	0.18 (0.11)	0.18 (0.08)	0.15 (0.08)	2.11	0.13
	Hold Ratio								
	Dominant	1.70 (0.76)	1.51 (0.52)	1.90 (0.83)	2.09 (1.28)	2.15 (1.36)	2.29 (1.08)	0.46	0.64
	Non-dominant	1.83 (0.79)	1.62 (0.63)	2.12 (0.84)	2.37 (2.38)	2.17 (1.04)	2.42 (1.09)	0.39	0.68
	Lift Duration (ms)								
	Dominant^a^	277.78 (160.24)	309.74 (129.72)	299.67 (96.32)	290.01 (86.93)	235.11 (66.37)	253.43 (86.31)	0.41	0.66
	Non-dominant	330.74 (186.32)	359.03 (150.55)	252.93 (99.38)	295.65 (123.32)	253.56 (99.23)	255.26 (102.75)	0.42	0.66
Purdue Pegboard	No. of pegs								
	Dominant	14.57 (1.55)	15.21 (1.76)	15.20 (1.78)	15.07 (1.90)	15.20 (1.93)	16.13 (1.41)	1.55	0.22
	Non-Dominant	14.00 (2.04)	14.21 (1.53)	13.73 (2.25)	14.57 (1.60)	14.13 (2.07)	14.67 (1.76)	0.31	0.73
Grooved Pegboard Test	Time to complete								
	Dominant	56.87 (7.92)	55.47 (7.66)	58.14 (11.03)	55.07 (6.82)	56.02 (7.84)	53.59 (6.36)	0.18	0.83
	Non-Dominant	62.37 (6.07)	60.77 (8.34)	61.31 (7.78)	59.09 (5.23)	62.40 (13.05)	61.59 (11.28)	0.26	0.77

^a^Model includes ‘history of computer gaming’ variable as a random effect

*p ≤ 0.05

*p*-values calculated using linear mixed model analysis

There was a significant improvement over time for the GPT (Dominant hand) outcome (F_1, 43_ = 5.215, *p* = 0.027), also on Log_10_ transformed data. There were no other significant within-group changes.

## Discussion

After a period of sensory awareness training, or motor practice, there were few significantly different outcomes evident between either of the experimental groups and the control condition. This included both psychological outcomes as measured by the IMI and measures of dexterity. This suggests that there may be little benefit from sensory awareness training or additional motor practice over the current scalpel teaching regime employed at either of the university programs.

The only variable for which there was a significant group difference over the experimental period was Preload Duration of the non-dominant hand for the grip-lift task. Preload Duration represents the duration of time between when a force is applied to the manipulandum and when the manipulandum leaves the supporting surface. A longer period is indicative of a poorer strategy or performance and therefore poorer dexterity. The assumption of the grip lift task is that it represents the interaction between afferent and efferent stimuli [8]. The main reason for this significant outcome was due to a larger improvement (decrease in mean time) displayed by the control group, relative to a smaller deterioration (increase in mean time) for both the sensory awareness and motor groups’ performance. However, it should also be noted that there were significant differences between the groups at baseline testing (F_2,39_ = 4.008, *p* = 0.026), with the control group performing considerably poorer than both of the experimental groups which may have contributed to the relative improvement or deterioration over the testing period consistent with the ‘law of practice’ where the rate of improvement is related to the amount left to improve [6, 20].

There could be a number of other reasons to explain the lack of between-group differences on the psychomotor outcomes within this study. Firstly, lower recruitment than anticipated resulted in only 44 participants overall suggesting the possibility of a type I statistical error. A post-hoc analysis undertaken on the dominant hand of the PP showed that an effect size of 0.29 was achieved thereby requiring a total sample size of 120 participants in order to observe a statistically significant change.

Secondly, 2 weeks may not have been sufficient time to see benefits of extra training (or indeed any training). As this is the first study investigating sensory awareness training in this context, we do not know how much time would be required to see a measurable change on the chosen tests. In a previous study [4], sensory awareness training of 40 min resulted in significant improvements on the PP and Grip-Lift task. However, this may be the consequence of immediate testing and does not represent lasting change. Undertaking retention testing, such as in this study may not have demonstrated similar change [21]. Savion-Lemieux and Penhune [22] found that the total number of practice sessions was not influential in motor learning; rather the distribution of sessions over time was the influencing factor. There was no significant difference between the study groups for the duration of intervention. Neither the number of sessions undertaken or time dedicated to training was significantly different between the Sensory or Motor groups. Whilst we cannot specifically analyse the distribution of sessions, the lack of difference for the duration of training or for the number of sessions suggests that this is unlikely to be very different between the groups.

Thirdly, the interventions may have lacked fidelity in their administration. A study by Breitenstein et al. [23] investigated intervention fidelity, identifying it as a particular issue not frequently considered with respect to intervention studies. For example, we only have self-reported data of participation in training and perhaps supervised practice would improve effectiveness by improving engagement (rather than the use of audio recordings and self-initiated practice). Further to this, the usual teaching practice at both sites (control) is likely to have high fidelity, providing increased motivation and high levels of feedback to the student. Thus additional training over and above this would not result in observable improvement.

Finally, the tests may not be sensitive to the changes induced in dexterity by the training. This may be the consequence of test elements which do not suitably represent improvement in the learned skill, or noise within the data produced by individual variability during the error-based learning period. The variability in individual performance in response to the intervention suggests there may be unidentified confounding factors, uncontrolled elements or that the training methods need to be more specifically tailored to the individual. This may include individual differences such as learning style, innate ability or state-like traits such as anxiety or motivation.

## Conclusion

The use of sensory awareness training, to target the sensory aspect of the afferent, cognitive and efferent triad in motor learning and performance, or additional motor practice did not provide a more effective alternative to learn scalpel skills compared with usual practice. Further research may be warranted using more engaged training, provision of supervision and greater participant numbers.

## Additional file

Additional file 1: Title: Grip-Lift test outcomes of interest. Provides an explanation and definition for each of the outcomes analysed as part of the grip-lift test. (ZIP 65 kb)

## Abbreviations

ATM: Awareness Through Movement
BIC: Bayesian Information Criterion
dGF/dt: Rate of Change of Grip Force over time
dLF/dt: Rate of change of Lift Force over time
DST: Dynamical Systems Theory
GF: Grip Force
GF:LF: Grip Force to Lift Force Ration
GFavg: Average Grip Force
GFmax: Maximum Grip Force
GFsd: Standard Deviation of Grip Force
GPT: Grooved Pegboard Test
IMI: Intrinsic Motivation Inventory
LF: Lift Force
LFDn: Lift Force Duration
LFmin: Minimum Load
PDn: Preload Duration
PP: Purdue Pegboard
QUT: Queensland University of Technology
SMS: Short Message Service
UniSA: University of South Australia
