# Supplementary material for: A randomised controlled trial of sensory awareness training and additional motor practice for learning scalpel skills in podiatry students
Source: BMC Med Educ. 2016 Dec 5;16:309. doi: 10.1186/s12909-016-0817-8 (PMC5139119; doi:10.1186/s12909-016-0817-8)
Supplement: Additional file 1: — Title: Grip-Lift test outcomes of interest. Provides an explanation and definition for each of the outcomes analysed as part of the grip-lift test. (ZIP 65 kb) [file 12909_2016_817_MOESM1_ESM.zip › 12909_2016_817_MOESM1_ESM/12909_2016_817_MOESM1_ESM.docx]

## Supplementary File One: Grip-lift test outcomes of interest.

- **Preload duration (PDn)** (ms) (Figure S1: T_1_-T_2_): time between onset of Grip Force (GF) and onset of positive Lift Force (LF). This may be preceded by a negative LF if the manipulandum is pushed into the supporting surface. A longer period corresponds with a clumsy lift strategy.
- **Minimum load (LFmin)** (N): maximum downwards force applied during PDn if the object was pushed into the surface as part of the lift phase. The greater the force the poorer the technique when attempting to grip and lift the manipulandum.
- **Maximum grip force (GFmax)** (N): peak GF reached during the lift phase. Optimum GF is the lowest force applied without slippage occurring and is made up of the force required to overcome LF plus a margin of error. Therefore, the higher the GF applied the greater the margin of error, and considered a poorer performance.
- **Grip force to Lift force ratio (GF:LF)**: ratio of GF to LF at GFmax during the lift phase. A low ratio suggests a better scaling of required force and therefore a better grip-lift strategy.
- **Maximal cross-correlation:** the maximum correlation coefficient obtained when the rate of change of GF over time (dGF/dt) and rate of change of LF over time (dLF/dt) were cross-correlated using time-shifts of 2.5 ms. A cross-correlation value of one indicates a perfect response of the participant’s grip force regulation synchronous to the increase in lift force.
- **Time-shift** (ms): the incremental shift required for maximum cross-correlation of dGF/dt and dLF/dt. This is calculated using 2.5 ms increments as outlined above until maximum cross-correlation is obtained. The time-shift is an indicator of whether the grip was primarily anticipatory or reactive. A positive time-shift indicates an anticipatory strategy and conversely a negative time-shift indicates a reactive strategy.
- **Lift Duration (LFDn)** (Figure S1: T_2_-T_3_): The time from which LF onset occurs until the force reaches within 98% of maximum LF. Previous studies have found poorer strategies to be associated with a longer lifting duration.
- **Average grip force (GFavg)** (N): The average GF during the hold phase. A higher average force is indicative of poor scaling of force relative to the weight and therefore poorer lift strategy.
- **Standard Deviation of Grip Force (GFsd)**: The standard deviation of the GF during hold phase, i.e. how much the GF varies whilst trying to maintain the manipulandum at the required height. Less change in force over this time results in lower standard deviations, indicating better control.
- **Hold ratio**: The average ratio of GF to LF as the object is held stationary during the hold phase. If the participant uses greater force to hold the manipulandum stationary, suggesting a less efficient technique, then the ratio increases.

Figure S1: Actual example of a lift with indicative time points used for variable calculation

(based on a diagram by Duque et al.)

Duque J, Thonnard JL, Vandermeeren Y, Sebire G, Cosnard G, Olivier E: **Correlation between impaired dexterity and corticospinal tract dysgenesis in congenital hemiplegia**. *Brain* 2003, **126**(Pt 3):732-747.
