# Supplementary figures and images for: A randomised controlled trial of sensory awareness training and additional motor practice for learning scalpel skills in podiatry students
Source: BMC Med Educ. 2016 Dec 5;16:309. doi: 10.1186/s12909-016-0817-8 (PMC5139119; doi:10.1186/s12909-016-0817-8)

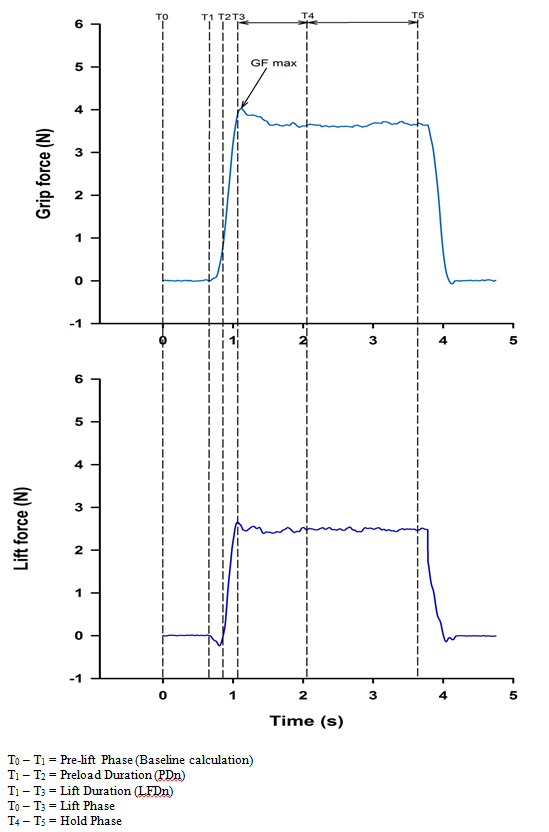

Supplement: Additional file 1: — Title: Grip-Lift test outcomes of interest. Provides an explanation and definition for each of the outcomes analysed as part of the grip-lift test. (ZIP 65 kb) [file 12909_2016_817_MOESM1_ESM.zip › 12909_2016_817_MOESM1_ESM/BMCmedFig3R2.png]
